# Nurses' Pain Assessment and Attitudes: A Qualitative Study in a Neurointensive Care Unit

**DOI:** 10.1111/nicc.70408

**Published:** 2026-03-12

**Authors:** Janni Kristina Voldbjerg, Suzanne Forsyth Herling, Birgitte Nørgaard

**Affiliations:** ^1^ Department of Neuroanaesthesiology – The Neuroscience Centre Copenhagen University Hospital Copenhagen Denmark; ^2^ Department of Clinical Medicine, Faculty of Health Sciences University of Copenhagen Copenhagen Denmark; ^3^ Department of Public Health University of Southern Denmark Copenhagen Denmark

**Keywords:** attitudes, nurses, pain assessment, prejudices, substance abuse

## Abstract

**Background:**

As analgo‐sedation gains prominence, a significant portion of adult ICU patients—including those in neurointensive care units (neuro‐ICUs)—remain awake and conscious throughout their stay. Despite this, neuro‐ICU patients frequently experience moderate to severe pain, highlighting the critical need for enhanced pain assessment.

**Aim:**

This study aimed to explore nurses' assessments and attitudes towards pain in critically ill patients in the neuro‐ICU.

**Method:**

A qualitative, exploratory and inductive study was conducted, inspired by Ricoeur's phenomenological‐hermeneutic approach. Data were gathered at a neuro‐ICU in Copenhagen, Denmark via four focus group interviews and five semi‐structured individual interviews with nurses. The Consolidated Criteria for Reporting Qualitative Research guided the reporting process.

**Findings:**

Four key themes were identified in the qualitative analysis: *Subjective pain perception*, *exclusive nursing responsibility for pain assessment*, *continuity for interpreting lived pain* and *attitudes undermining pain care in the neuro‐ICU*. Nurses viewed neuro‐ICU patients with brain injury as unable to self‐assess pain due to cognitive and communication deficits, irrespective of sedation level, leaving pain assessment dependent on the nurses' interpretive judgement.

**Conclusion:**

Nurses in neuro‐ICU found pain assessment challenging due to impaired communication and fluctuations in consciousness, independent of sedation level. Nurses' dependence on physical parameters and tacit interpretive judgement placed clinical decision‐making in their hands when medical input was absent. Under these conditions, continuity of care became vital for interpreting patients' subjective pain cues. This need for continuity was further amplified by nurses' perception of holding sole responsibility for pain assessment in the neuro‐ICU.

**Relevance to Clinical Practice:**

The findings emphasize the need for structured pain assessment approaches that combine standardized tools with nurses clinical judgment in analgo‐sedated neuro‐ICU patients. Continuity of care appears essential for recognizing individual pain expressions and supporting consistent clinical decision‐making. Stronger interdisciplinary collaboration may reduce the burden on nurses and improve shared responsibility for pain management. Reflective practice and education are needed to address implicit biases, particularly toward patients with substance use histories. Together, these initiatives may reduce variation in analgo‐sedation practices and better protect patient comfort.

## Impact Statements

- What is known about the topic
  ○ Delirium is a common and serious complication among ICU patients worldwide.
  ○ International guidelines highlight the gaps in evidence for pain assessment, especially for neuro‐ICU patients.
  ○ Neuro‐ICU patients can exhibit atypical signs of pain challenging pain assessment.
- What this paper adds
  ○ Nurses in the neuro‐ICU find pain assessment challenging Nurses’ reliance on physical indicators and tacit interpretive judgments guide their pain assessment
  ○ Nurses paternalistic preunderstanding limit their access to the patients’ lived and expressed signs of pain.

## 1 Introduction

Physicians and nurses provide multidisciplinary care to improve outcomes for critically ill patients with complex neurological conditions in neurointensive care units (neuro‐ICUs) [1]. Consequences of complex neurological conditions are that critically ill patients are unable to communicate their pain, why nurses are obliged to rely on systematic clinical observations and validated assessment tools to guide pain assessment [2]. To enhance neuro‐ICU treatment and comfort, analgo‐sedation (a combination of analgesia and sedation allowing the patient to remain conscious) is increasingly favoured [3]. And over the last 15 years, there is a growing number of neuro‐ICU patients that remain less sedated during their ICU stay [4, 5, 6]. However, there are still reports that suggest that critical ill patients in the neuro‐ICU are experiencing moderate to severe pain [7]. Reasons for pain are numerous and may be linked to the trauma, as a neuro‐ICU stay is often preceded by an accident or trauma [8]. Other reasons for pain are nursing care, in terms of suctioning, repositioning and mobilising the critically ill neuro‐ICU patients [9, 10] as well as fatigue and weakness caused by bedrest in general [10].

When looking at reasons of accidents are linked to substance abuse; for young men it is almost half of cases [11]. Substance abuse complicates pain assessment due to altered pain perception, opioid tolerance and slurs the clinical picture by possible withdrawal symptoms [11].

### 1.1 Background

It has been previously reported that pain assessments conducted by nurses appear unsystematic and haphazard [12]. Moreover, international guidelines on pain highlight the gaps in evidence for pain assessment, especially for neuro‐ICU patients [13]. Earlier neuro‐ICU patients were excluded from trials due to their fluctuations in consciousness caused by severe brain injury, which made it difficult for them to consent to research [14, 15, 16]. Therefore, knowledge of pain assessment in neuro‐ICU patients may be based on research conducted with general ICU patients [17]. One very cited study shows that neuro‐ICU patients can exhibit atypical signs of pain, challenging standard assessment and exposing deficiencies in validated screening tools [18]. However, evidence‐based nursing requires the integration of different types of knowledge, where tacit experience must be made explicit so it can be shared, evaluated and developed as valid evidence [19].

### 1.2 Aim

This study aimed to explore nurses' pain assessments, their attitudes towards pain and their tacit clinical experiences when caring for analgo‐sedated neuro‐ICU patients.

## 2 Methods

### 2.1 Study Design

A qualitative, exploratory and inductive study design was applied, based on semi‐structured focus group interviews and individual interviews with nurses employed in a neuro‐ICU. Ricoeur's phenomenological‐hermeneutic philosophy was chosen as an interpretive framework that moves beyond mere description to uncover deeper meanings through a dialectical interplay of explanation and understanding [20, 21]. Unlike other qualitative approaches, Ricoeur emphasises the necessity of critical distanciation—a deliberate analytical distance between the text and the interpreter—which prevents immediate subjective bias and enables a more rigorous interpretation of lived experience [21, 22].

This theoretical lens was consistently applied throughout the analytical process. Interview transcripts were treated as texts rather than nurses' narratives, aiming and providing an interpretation that are independent of our preunderstandings [21]. The analysis followed Ricoeur's theory of interpretation, beginning with naïve reading to grasp an initial holistic understanding, progressing to structural analysis to identify patterns and thematic structures, a culminating in critical interpretation that revealed ethical and existential dimensions embedded in the narratives [21].

The concept of triple mimesis further guided interpretation by acknowledging how nurses' accounts are prefigured by prior experiences and cultural norms (Mimesis I), configured through narrative discourse (Mimesis II) and refigured in the researcher's comprehension (Mimesis III) [22]. This process allowed the study to move systematically from surface‐level meaning to a deeper interpretive understanding [20, 21, 23]. The use of Ricoeur's framework is particularly significant because it does not merely describe nurses' experiences but situates them within a broader ethical and epistemological context [21]. By uncovering how clinical judgements regarding pain assessment are shaped by both explicit knowledge and tacit experiential dimensions, this approach offers a more nuanced understanding of decision‐making processes than conventional qualitative methods [22]. This framework enables systematic movement from naïve understanding to critical interpretation [23].

### 2.2 Setting and Participants

Data were collected from nurses working in a high dependency ICU (level III) in Copenhagen, Denmark, comprising 16 neurological/neurosurgical beds and staffed by 130 nurses. Nurse–patient ratios were maintained at 1:1 or 1:2 for all shifts. Patient allocations were determined by clinical competencies and educational background and the needs for both nurses and medical doctors (MD's). The average ICU length of stay was 5 days, with an annual patient turnover of approximately 1.000 admissions. The department protocol instructs pain assessment every shift and on demand, requiring nurses to use validated screening tools, such as visual analogue scale (VAS), numeric rating scale (NRS) or critical‐care pain observation tool (CPOT), in line with international ICU guidelines [13]. We included registered nurses (RN) and both certified and non‐certified intensive care nurses working in the neuro‐ICU.

Excluded were nurses who were not native Copenhagen, Denmark speaking or had less than 4 months of employment, as we wanted participants with experience particularly in pain assessment and management within the intensive care setting [2].

A total of 20 eligible focus group participants were invited by the first author and ICU staff nurse manager and accepted the invitation; four participants were unable to participate, after all. First, we conducted four focus group interviews, including a total of 14 nurses, followed by five individual interviews with participants re‐selected from the focus group sample for deeper exploration of their views and perspectives [24].

All participants were selected through purposeful sampling with maximum variation [25] on gender, age, educational level and clinical experience in pain assessment within the unit, enabling diverse experiences and comprehensive descriptions. The first sampling was for focus group interviews. The second sampling consisted of participants who had given controversial or key statements during the focus groups. The first author established and moderated all interviews after obtaining written consent from the participants. Focus group interviews were chosen to provide a structured setting for participants to share insights, challenges, perspectives and refine collective understanding [24]. (The participants are presented in Table 1).

**TABLE 1:** Demographics and number of interviews with RNs.

Type of data	Demographic data	Inclusion criteria	Participants	Number of interviews	Average interview duration
Focus group interviews	Age: 27–59 years RN experience: > 1–29 years RN experience in neuro‐ICU: 4 months–17 years	RN > 4 months employment in neuro‐ICU Speaking and writing the main language fluently	14 (12 women and 2 men)	4	45–55 min
Single in‐depth interviews	Age: 27–47 years RN experience: 2–20 years RN experience in neuro‐ICU: 1½–17 years		5 (women)	5	45–55 min

Abbreviations: neuro‐ICU, neurointensive care unit; RN, registered nurse.

### 2.3 Data Collection

The research group created and modified the interview guides based on the literature and clinical experience. Both started with a short narrative to prime the mindset of the participants [22].

Interviews were conducted from November 2023 to July 2024 in an office near the neuro‐ICU. The first author (who identifies as female and is an experienced ICU nurse, interviewer and co‐worker to all participants) moderated all focus group and individual interviews using an interview guide. (See Table 2).

**TABLE 2:** Examples from the interview guide in focus group and single in‐depth interviews.

Interview guide used in focus group interviews
Case A: Discuss the situation for the fictive patient John. John's condition right now: He has a TBI and has difficulty expressing himself, as he is tracheostomized and still without a speech valve. He is currently out of sedation. John has mood swings, and he can appear irritable.
How would you rate John's pain over the course of the day? How can you observe pain if John can't express himself? When assessing pain throughout the day, would you consider John to be able to sleep despite severe pain? Do you think that if John could speak up, he would assess pain intensity differently from the HCP?
What if the HCP and the patient assess pain intensity differently? Who do you think can give the most accurate assessment of pain intensity: the patient or the HCP? If your patient is a recovering addict or current addict, do you consider this in your pain assessment?
**Interview guide used in‐depth single interviews**
How would you assess pain in neuro‐ICU patients? What do you think about the following quote: ‘I kind of forget [that it is] my assessment of the patients, that it is actually subjective, [so I will] bring my own preconceptions into [the pain assessment]’? How do you understand pain? What do you think about whether pain can be physical, psychological and/or social?
What do you think about the following quote: ‘[If] they are fishing for a painkiller’? Do you consider history of substance abuse when caring for a patient? What do you think about the following quote: ‘They say they are in pain, those who have had a taste for morphine, and then I think [it] is difficult with [them]’? Some nurses mention it's important ‘not to start him on a new addiction’. How do you think of your role as a nurse in relation to the above quote?

Abbreviations: HCP, healthcare professional; neuro‐ICU, neurointensive care unit; TBI, traumatic brain injury.

Prior to the first interview, the preunderstandings of the moderator were documented in a separate file. Initially, two pilot focus group interviews were conducted, followed by minor alterations of the interview guide by clarifying and shortening to improve reliability [26, 27]. All interviews were transcribed verbatim by the first author. A fellow researcher served as observer during focus group interviews, noting activities and nonverbal interactions. Notes had descriptive details (e.g., setting, interactions and atmosphere) and reflective insight (e.g., lessons learned) used for the initial analyses.

Each focus group and single in‐depth interview was conducted on audio within 45–55 min, as agreed upon, because of the nurses' workload in the neuro‐ICU. This study is reported in adherence to the Consolidated Criteria for Reporting Qualitative Research (COREQ) checklist [28].

### 2.4 Data Analysis

To operationalise the phenomenological‐hermeneutic analysis method inspired by Ricoeur we followed the three steps (1) naïve understanding, (2) structural analysis and (3) critical analysis by Dreyer and Pedersen [23]. All interviews were treated as a unified text and analysed utilising Ricoeur's principles of interpretation to uncover a deeper meaning [22]. The analysis was based on a mix of data from both types of interviews, and relevant quotes were captured from both sources. Initially, a naïve reading provided a preliminary holistic understanding. Subsequently, structural analysis moved from understanding of ‘What it says’ to ‘What it speaks about’ [23]. Although we complied with COREQ importing guideline, we had not conducted a member check as Ricoeur, would have considered having created a new text [23]. Finally, a critical analysis and discussion created these themes and deepened the analysis on relevant research literature and placed it in a broader interpretive framework [20]. Application of the analysis steps, as illustrated in Table 3, demonstrated the progression from text segments to thematic formulation.

**TABLE 3:** Example of the interpretation steps in data analysis.

Citation/What it says	Meaning/What it speaks about	Theme
*‘What you have in your baggage [and] where you are in your life can affect pain. If you're already in an intensive care unit, like here with a bleed in your head, and you're scared, and you're anxious, and you may not have the greatest social resources. Then you might also feel a little more pain, or you feel pain quickly in advance to those who are more resourceful.’*	When trying to understand and interpret the pain experience of this neuro‐ICU patient, it is crucial for the HCP to consider all these individual factors that may influence the patient's pain perception and how the patient deals with the subjective experience of pain from previous experiences.	‘Subjective pain perception’.

Abbreviations: HCP, healthcare professiona; neuro‐ICU, neurointensive care unit.

The research team of three ICU nurses had different distance to the theme. Reflexivity was maintained throughout the analysis by documenting preunderstandings, revisiting this understanding during analysis, keeping reflexive notes and engaging in iterative dialogue within the research team. Interpretations and emerging themes were critically discussed to minimise bias an ensure transparency. An audit trail supported credibility by tracing the progression from meaning units to themes [23].

### 2.5 Ethical Considerations

The study was approved by the management of the department and Copenhagen, Denmark location of University (ID 12.109); Approval Date: December, 20, 2023. According to national legislation, formal ethical approval for qualitative studies is not needed [29]. All participants provided both written and verbal consent after being informed about the study's purpose, intent and data collection procedures. Furthermore, all participants were informed that their participation was voluntary, that they could withdraw their consent at any point without consequences, and that their statements would be treated confidentially and anonymised in the final publication.

## 3 Findings

Four themes were identified, indicating that assessing pain in neuro‐ICU patients can be challenging due to fluctuations in consciousness for both awake and sedated patients. The themes were as followed: (1) subjective pain perception, (2) exclusive nursing responsibility for pain assessment, (3) continuity for interpreting lived pain and (4) attitudes undermining pain care in the neuro‐ICU.

### 3.1 Subjective Pain Perception

Nurses considered pain to have both a physical and a psychological side, which mixed their experiences substantially: ‘The pain can be physical, and it can also be psychological, where you feel the pain is present, but in reality, it isn't pure pain*’* (Participant 9).

The nurses' distinction between physical and psychological pain indicating a dichotomous understanding of pain—where psychological pain was not necessarily considered as reel pain in the patients. Some nurses considered only physical parameters as essential to appraise after the acute phase of pain in neuro‐ICU patients:

*I think you can divide it into, for example, physical pain and psychological pain. What I mostly deal with is physical pain, and that is probably the most tangible for me as a nurse to alleviate here and now. Whereas the psychological pain, which I also see our patients experiencing, is something that extends over a longer period and perhaps [is] not the acute pain in the critical phase [that we] deal with, roughly speaking, I think* (Participant 11).

Physical pain appeared as an immediate phenomenon—urgent and concrete—whereas psychological pain unfold as a persistent experience that transcended the critical phase. The nurses acknowledged the complexity of pain and the need to consider both physical and psychological aspects in their care and the pain assessment, as well as recognising that perception of pain was individual:

*Then it's a form of subjective pre‐understanding because I have my pre‐understanding. I have my experience, but it is still subjective because what I understand by pain is different from what you understand by pain* (Participant 1).

Other participants spoke of the psychological influence on patients' pain perception and suggested bereavement as a pain trigger. Again, the examples were explained in relation to the nurses' own life experiences introducing events or illnesses that would produce pain, such as childbirth and divorce, as in this quote:

*I mean, I think a birth would be a painful experience, yes, a migraine attack is a painful experience, a broken arm is a painful experience. That would be physical pain. I also think [that] a death is a painful experience, yes [and a] divorce, yes* (Participant 6).

This interpretative dimension resonated with the nurses' own reflections, as they described pain as subjective and shaped by their experiences and preconceptions. Their personal backgrounds influenced how they interpreted pain, leading to variations in assessment and management. Nurses demonstrated egocentric interpretation bias as they assessed pain under strong influence of their own experiences. This highlighted the role of preconceptions and subjectivity and aligns with the idea that one's prior understanding and context influences their pain assessment and interpretation, as stated by this participant: ‘But that is my experience. Yes, and I also think we differ in when we want pain relief*’* (Participant 1).

### 3.2 Exclusive Nursing Responsibility for Pain Assessment

The nurses described feeling solely responsible for the pain assessment, whereas the role of MDs was to assist by prescribing medication to ensure adequate pain treatment. Nurses did not question this division of responsibility between the different healthcare professionals (HCPs) and spoke of how the MDs relied on and acted on the nurses' assessments; however, they would have preferred a second opinion on their pain assessment because they are worried, that their assessment might be favoured. Nurses expressed that adequate pain relief was very important to ensure patient comfort:

*I think it is the MD who should prescribe the right pain treatment for the patient. So, they also need to assess before they prescribe the right one. But they do it in collaboration with the nurses, and what I have experienced is that the MD asks, how do you perceive the patient's pain. So, I also think the nurses have the greatest responsibility for assessing the pain* (Participant 9).

From a Ricoeurian perspective, this highlights the interplay between nurses and MDs in pain management and reflects an interpretative dimension of care, clinical judgement extended beyond technical action to a shared sense of responsibility. Within this dynamic, nurses often perceived themselves as bearing the greatest responsibility for alleviating suffering, as their subjective experiences and proximity to the patient shape both assessment and response. Dialogue with MDs mediated these interpretations, enabling a more comprehensive and ethically attentive approach to pain relief:

*The responsibility for ensuring it [pain assessment] is entered into the system [Electronic Patient Journal and] that the pain is assessed lies with the nurse. It is the nurse's responsibility. That is definitely my opinion* (Participant 1).

Although nurses recognised their professional responsibility to record and document pain scores they implied, that it rarely happened as pain assessment was not always requested by the MD in charge of the patient. It was suggested that validated pain assessment tools would be helpful used by both professionals. Nurses emphasised on careful documentation illustrated how clinical responsibility extended beyond isolated interventions and became an interpretive act shaped by time, attention and the ethical obligation to ensure patient comfort.

### 3.3 Continuity for Interpreting Pain

The nurse's assessment of pain is significantly influenced by the patient's level of consciousness and ability to communicate, as the perceived credibility of the assessment is higher when direct communication is possible. However, this ideal is complicated in neuro‐ICU settings, where patients often inhibit such interactions:

*No, but the credibility is different if it is an awake patient I can ask [and communicate with]. Yes, because then it is not my assessment of it. Okay, and then again, as I said, our patients are brain‐injured, so it also depends on how awake the patient is* (Participant 1).

From the nurse's perspective, continuity of care is crucial for optimising pain relief and understanding neuro‐ICU patients' subjective pain experience, especially when communication is challenging. The nurses believed that continuity of care—ongoing presence—reflects a temporal and relational dimension of care and enables nurses to interpret subtle changes in pain and comfort over time. Nurses proposed continuity among HCPs as a solution to give patient comfort and followed by careful documentation in patient records: ‘It could be something as simple as continuity in the care staff. For example, the same MD, the same nurse, and so on*’* (Participant 6).

*To create some continuity [with] this, we also need to hand over shifts. Having these shifts where you hand over and pass on [knowledge] to your colleague, it [is] good to have it documented… so it can be done a bit more systematically* (Participant 11).

From a Ricoeurian perspective, documentation was more than a technical record—it was rather a narrative trace that supported shared understanding and responsibility in action with patients' reduced level of consciousness and limited ability to communicate. Nurses thereby took on a central interpretive role, highlighting the complexity of pain assessment.

### 3.4 Attitudes Undermining Pain Care in the Neuro‐ICU

Pain assessment in patients with a history of substance abuse exposes a tension between trust and suspicion, where stigmatisation and fear may influence clinical recognition. From a Ricoeurian perspective, this process constituted a relational and interpretive responsibility, obliging clinicians to move beyond mere clinical observations and towards an ethical understanding of the patient's suffering.

Considering the possibility of malingering exposed, the inherent tension between trust and suspicion and underscored that clinical decision‐making must still safeguard patient comfort. Pain assessment in patients with a history of substance abuse illuminated the interpretive complexity of clinical assessment. Viewed through a Ricoeurian lens, nurses' fear of overmedicating and unintentionally sustaining substance abuse exposed a tension between care and control, where clinical decisions unfolded as negotiated responses to the ethical uncertainty of another's suffering.

Amid nurses' fear of misjudging pain in neuro‐ICU patients with current or past substance abuse, they acknowledged that preconceptions and attitudes might contribute to both under‐ and overtreatment with opioids. Nurses were concerned that patients were malingering and asking for more pain medication that they clinical observative needed or that patients claimed less pain to avoid opioid in fear off relapsing or stigmatisation. Critically ill patients in neuro‐ICU with a history of substance abuse might potentially experience significant pain during their ICU stay to avoid relapse and stigmatisation:

*There can be many reasons why they say they do not want anything. We have also had some who previously struggled with addiction and were afraid of relapsing if they take something, so they prefer to endure the pain* (Participant 1).

This concern is further compounded by the ongoing opioid epidemic in Western societies. Nurses indicated that their clinical decision‐making concerning their administration of opioids was influenced by this epidemic:

*We live in a society where opioids are taboo. It is a really difficult patient [with a history of substance abuse], I think. Because partly… there is a risk that we underestimate …, I think, prejudice, [that] they might say, I am in so much pain. So, I might assess it lower, and then they might actually be in so much pain, because I think they probably want some morphine or something* (Participant 6).

The nurses expressed the need to recognise and address these biases to ensure objective and fair pain perception and establish regular pain relief in patients with current or past substance abuse: ‘I try to be very mindful of setting that aside’ (Participant 7).

Attitudes could further influence decision‐making, particularly in high‐pressure ICU settings. Consequences, pain management might be inadequate—not because of clinical necessity, but due to prejudice, uncertainty and risk‐averse behaviour—which could compromise patient comfort.

## 4 Discussion

This study aimed to explore nurses' pain assessments, their attitudes towards pain and their tacit clinical experiences when caring for analgo‐sedated neuro‐ICU patients. By applying Ricoeur's phenomenological‐hermeneutic framework, the nurses' narratives became meaningful for pain assessment in the neuro‐ICU, where understanding and decision‐making were closely linked. Our findings showed that pain perception was inherently subjective and could not be captured through objective measures alone. Ricoeur's phenomenological‐hermeneutic framework underscored that pain assessment extends beyond observation, emphasising the interpretive work through which nurses constructed meaning in complex clinical contexts [22]. The interpretation of patients' pain was therefore not neutral but shaped by nurses' experience, intuition and ethical judgement—a perspective consistent with Ricoeur's theory of interpretation [23]. Neuro‐ICU patients were often intubated or have impaired communication skills, and therefore, it was the nurses' interpretation of the patient's pain experience that was crucial for decision‐making [2]. Findings revealed that psychological and social indicators were not always recognised as ‘pure’ pain experiences—a dichotomous understanding when pain experiences only focus on biological causes of disease as objective measurements [30]. This limitation posed challenges in integrating psychological and social dimensions into pain assessment, despite increasing recognition of their importance [30]. Nurses also demonstrated egocentric interpretation bias as they assessed pain under strong influence of their own experiences. Egocentric interpretation bias arises from precision‐weighted self‐related predictions, meaning that greater certainty about one's own state intensifies projection onto others [31]. Egocentric bias is rooted in self‐related predictive mechanisms [31]. Although broader cognitive bias could hinder evidence‐based decision‐making [32]. Egocentric interpretation bias is common cognitive bias, which operates largely without conscious intent [31]. This raised critical questions as to whether similar egocentric interpretation bias could influence nurses' pain assessment, potentially compromising the quality of care and patient comfort. From an ethical perspective, it posed challenges in terms of patient safety if nurses' subjective assessments leaded to undertreatment of the patient pain experience because nurses should carefully balance pain relief and avoid harm to the patient, where the consequences of misjudgements could be serious in the neuro‐ICU, based on Ricoeur's ethical intention as discussed by Russo [22], a perspective consistent with Ricoeur's phenomenological‐hermeneutic approach, and the concept of distanciation [23].

Nurses in the study recognised their responsibility for pain assessment, and this sense of obligation to promote patient‐centred care could induce pressure, especially when rapid decisions had to be made with limited information. Nurses noted the limited information and suggested using a validated tool, such as CPOT, for noncommunicative patients [13].

Nurses emphasised that continuity of care was crucial for recognising and interpreting neuro‐ICU patients' pain and changes in their condition. Nurses tended to rely on behavioural cues, physical indicators and clinical judgement to guide pain assessment in these cases [2]. Nurse–patient communication was further complicated if the patients were mechanically ventilated, as they were vulnerable due to fatigue, misunderstandings and misinterpretation of signals [33]. However, nonverbal signs such as grimacing or restlessness could have originated from neurological agitation rather than pain, thereby increasing the risk of misinterpretation [18]. Frequent handovers disrupted narrative continuity, fragmenting nurses' understanding of patients' pain and weakening decision‐making—a pattern that challenges Ricoeur's view of continuity as central to meaning‐making [23]. Structural barriers in the organisation, such as lack of continuity, can weaken the relationship and thus the quality of pain assessment [34]. Nurses in the study generally referred to neuro‐ICU patients as ‘brain‐injured’ and assumed they were unable to self‐assess pain due to cognitive and communicative impairments, regardless of sedation level. This reflected a paternalistic attitude, a pattern likewise identified in previous studies of general ICU nurses' pain perception [35].

The ongoing opioid epidemic may have shaped nurses' attitudes towards pain assessment by linking local practice to global discourses. Evidence indicates that concerns about opioid harm influence prescribing behaviour and can contribute to pain undertreatment [36]. This suggests the presence of implicit bias that may contribute to the undertreatment of pain in ICU patients with substance abuse histories [37]. Specifically, when patients with substance abuse histories are perceived as more likely to be stigmatised by nurses than other patients, this may distort pain assessment [37]. Stigmatisation refers to the process by which individuals or groups are ascribed negative stereotypes, moral judgements or biased assumptions that shape interactions and may lead to inequitable treatment [38].

Attitudes, in Ricoeur's view, are embedded in cultural and institutional narratives rather than existing in isolation [21]. Examining these narratives revealed how pain assessment unfolded a negotiated balance between alleviating suffering and maintaining clinical control. The global opioid crisis has intensified the tension between effective pain management and fear of addiction, contributing to pain stigmatisation and ethical challenges for nurses and patients who may refuse treatment due to stigmatisation [36]. Even before the pandemic, the prolonged use of opioids and sedatives in ICU patients had raised concerns about withdrawal symptoms [16, 17]. Global concerns about opioid addiction may affect adherence to analgo‐sedation protocols, requiring nurses to balance adequate analgesia with dependency risks in noncommunicative ICU patients [2, 36].

### 4.1 Implication for Practice and Ethics

The next step is to support nurses' interpretive capacity in neuro‐ICU pain assessment by integrating validated tools and counteracting egocentric bias and paternalistic preunderstanding. Education should rest on nonpaternalistic principles and strengthen evidence‐based practice to improve pain relief. This includes guidance on opioid use in the neuro‐ICU and stigma‐free care for patients with current or past substance abuse histories.

### 4.2 Limitations

This was a single‐centre study with a relatively small number of participants, which restricts the transferability of findings. However, the total number of participants (*n* = 14) was deemed sufficient to ensure information power [39]. According to Malterud et al., the strength of information should be seen in relation to the quantity and quality of data rather than the number of participants in qualitative research [39]. The participants provided rich descriptions that are in line with international research, which strengthens the credibility of this study.

Additionally, the dual role of the researcher as both co‐worker and interviewer may have positively influenced participants' openness. But social desirability may have influenced the interviews negatively and affected nurses' abilities to express themselves. The interviews were composed to reflect variation in gender, age, education and clinical experience, which is a strength; however, further research across multiple sites is needed to explore transferability to other settings.

## 5 Conclusion

The aim of the study was to explore nurses' pain assessments, their attitudes towards pain and their tacit clinical experiences when caring for analgo‐sedated neuro‐ICU patients. The findings revealed challenges in the clinical decision‐making. Nurses in the neuro‐ICU found pain assessment challenging due to impaired communication and fluctuating consciousness, independent of sedation level. Nurses' reliance on physical indicators and tacit interpretive judgements meant that clinical decision‐making rested with them in the absence of MDs' input. In this context, continuity of care became essential for interpreting patients' subjective pain cues, a need reinforced by nurses' perception of holding sole responsibility for pain assessment in the neuro‐ICU. Nurses prejudices a paternalistic preunderstanding—shaped by egocentric interpretation bias—limited their access to the patients' lived and expressed signs of pain and influenced the interpretive processes guiding clinical decision‐making. This undermined pain assessment and increased variation in analgo‐sedation protocols for patients with current or past substance abuse, highlighting the need to protect patient comfort during the opioid epidemic.

## Author Contributions

Conceptualisation, data curation, formal analysis, methodology: J.K.V. and B.N. Investigation, project administration, resources, visualisation: J.K.V. Supervision, validation, writing – review and editing: S.F.H. and B.N. Roles/writing – original draft: J.K.V., S.F.H. and B.N.

## Funding

The authors have nothing to report.

## Disclosure

Chat GPT was used to translate native language citations as well as language translation in the manuscript.

## Ethics Statement

Participating nurses consented voluntarily and the written consents are stored securely at University of Southern Denmark (SDU) (ID 12.109); Approval Date: December, 20, 2023.

## Conflicts of Interest

The authors declare no conflicts of interest.

## Data Availability

The data collected and analysed in the current study are not publicly available.
